# IPSCL: An Accurate Indoor Positioning Algorithm Using Sensors and Crowdsourced Landmarks

**DOI:** 10.3390/s19132891

**Published:** 2019-06-29

**Authors:** Beakcheol Jang, Hyunjung Kim, Jong Wook Kim

**Affiliations:** Department of Computer Science, Sangmyung University, Seoul 03016, Korea

**Keywords:** indoor positioning, magnetometer sensor, gyroscope sensor, landmark, crowdsourcing

## Abstract

Indoor positioning technology has attracted the attention of researchers due to the increasing pervasiveness of smartphones and the development of sensor technology, along with the increase of indoor time. Sensor technology, which is one of the most commonly used data sources for indoor positioning, has the advantage that sensors can receive data from a smartphone without installing any additional device. However, the readings of built-in sensors are easily affected by the surrounding environment and are even occasionally different from each other which adversely influence the accuracy of indoor positioning. Moreover, once an error occurs, it can accumulate because there is not any reference point in the sensor, only in indoor positioning. In this paper, we present an accurate indoor positioning technology, which uses smartphone built-in sensors and Bluetooth beacon-based landmarks. Our proposed algorithm chooses proper one between values of sensors alternately based on their characteristics. It exploits landmarks as the reference points of indoor positioning. It also allows individuals to add the location where they repeatedly detect the same and special beacon received signal strength indicator values as a crowdsourced landmark. Extensive experimental results show that our proposed algorithm facilitates the acquisition of accurate heading direction and coordinates of the user.

## 1. Introduction

Today, most people spend more than 80% of their time in an indoor space [1]. Various services and applications based on the indoor location of users have been developed and extensively used, and the importance of indoor positioning technologies is of increasing importance [2,3,4]. Positioning technologies use global positioning system (GPS) signals [5,6]. However, GPS signals are satellite signals and exhibit strong linearity. As such, they cannot penetrate the walls of a building and, therefore, cannot be used for indoor positioning [7,8]. One of the most common and accurate indoor positioning technologies is Wi-Fi-based fingerprinting, which consists of an offline training phase and an online estimation phase [9,10,11]. In the former, the indoor space of interest is divided into cells, and the received signal strength indicator (RSSI) values of the Wi-Fi signals [12] for each cell are collected, and a fingerprinting map is constructed. In the online estimation phase, users estimate their location based on the map. However, whenever Wi-Fi access points are added, modified, or removed or the interior features such as walls or even furniture are changed, the Wi-Fi signals must be distorted and the fingerprint map must be completely recreated. In this case, indoor positioning technologies without such limitations are required.

As the smartphone industry grows rapidly [13], one of the biggest milestones of this phenomenon is that many technologies can easily exploit the smartphone’s built-in sensors [14,15,16]. The built-in sensors are beneficial to indoor positioning technologies because they are universal and do not require additional devices. However, the readings of built-in sensors are affected easily by the surrounding environment and are even occasionally different from each other. Moreover, such errors can be continuously accumulated during indoor positioning [17,18]. For example, the gyroscope sensor and the magnetometer sensor among the built-in sensors can be used to measure a user’s heading direction for indoor positioning, which may be different from each other and occasionally from themselves. As a result, this error accumulates and the position of the user may become increasingly inaccurate.

The main objective of this paper is to propose an accurate indoor positioning technique estimating accurate heading directions and coordinates of users. Our proposed technique covers broad and necessary components of indoor positioning techniques. We propose our own technique, and show problems and potential roles of indoor various positioning components such as various sensors and landmarks through extensive experiments. Our proposed technique uses the built-in sensors in smartphones and Bluetooth beacon-based landmarks and is called indoor positioning with sensors and crowdsourced landmarks (IPSCL). IPSCL uses the gyroscope and the magnetometer sensors of the smartphone to track the position of a user in an indoor space. The readings of these sensors fluctuate and are occasionally different from each other; hence the heading direction of moving people as calculated using the sensors is inconsistent. IPSCL attempts to reduce the fluctuations of their values by making accurate choices between the values, based on their characteristics. When there is an error in such a sensor-based indoor positioning approach, it is not possible to correct the error because there are no reference points. IPSCL exploits the Bluetooth beacon-based landmark approach to solve this problem. The landmark is a datum point that is visible to serve as a reference by being differentiated from other points and exhibiting consistent patterns [19,20]. Our proposed technique also allows users to add the location where they repeatedly detect the same and special beacon RSSI values as a crowdsourced landmark. 

To evaluate our proposed technique, it was implemented in a Google Android platform [21,22] and we performed extensive experiments. We compare our proposed technique (sensors, predefined landmarks, and crowdsourced landmarks) with sensor only techniques, sensors, and predefined landmark techniques focusing on the heading direction error and distance error for the ground-truth reference path. We also extend our proposed technique to consider a typical situation that the indoor map is known and showed our proposed mechanism can be improved by ruling out unreachable area. The experimental results clearly demonstrated that the proposed technique provided accurate heading direction and coordinates of the user.

The remainder of this paper is organized as follows. In Section 2, we discuss related works. In Section 3, we describe the design of our proposed technique. We present the experimental results in Section 4. We present conclusions regarding the proposed system in Section 5. Finally, in Section 6, we discuss future research for the proposed system.

## 2. Related Work

Recent technologies involving indoor positioning rely on mobile data acquired via mobile sensors that accumulate errors, and they also exhibit various flaws because sensors are prone to fluctuations caused by the environment. Hence the performance of indoor positioning technology depends on how well it can be corrected and ease of removal of these errors to accurately provide the user’s location. Numerous approaches have attempted to solve this problem using secondary sensors or landmarks. 

BatTracker uses sensors to estimate the user’s position based on inertial data and corrects errors by measuring distance using acoustic signals reflected off nearby objects [23]. If there is a three-dimensional space consisting of a ceiling and two walls, it is possible to correct the position of the user based on the reflected sound waves in the space. However, BatTracker requires a three-dimensional medium, in which sound waves can be reflected. If the sound waves generated by the device are irregularly reflected off the medium, it is difficult for the sound waves to be detected. ApfiLoc [24] is another successful Calibration-free smartphone based indoor localization system that uses least-squares support-vector machine (LS-SVM) algorithm [25,26]. It fuses the augmented particle filter (APF) [27,28] and measurements from the inertial sensors of smartphones to provide localization with an error less than 2 m without relying on Wi-Fi support. However, it exhibits limitation in terms of obtaining precise heading estimations. SmartLight measures the position of a user by sensing a digital signal generated by a blinking light-emitting diode (LED) from an auxiliary light source sensor [29]. Not only can SmartLight use the light sensor of the device instead of the underlying auxiliary sensor, it can also reduce the cost of installing the infrastructure by changing the lighting in the room instead of installing the infrastructure to generate the light source separately. However, due to the nature of the technology for tracking location using the light source, it is impossible to measure the location if the device is not receiving light. This is a fatal drawback in the case of an emergency when there is a limited light source. visual simultaneous localization and mapping (vSLAM) is a technology used to create a visual landmark map using an object recognition algorithm based on a camera image from a mobile device [30]. Unlike other technologies, vSLAM has an advantage in that it does not require the installation of additional infrastructure or auxiliary devices. However, it relies on visual landmarks and due to the nature of the video as a landmark, the landmarks database is very large, especially in a large environment such as a shopping mall or a stadium. This makes it difficult to use in a big space or building where indoor elements change rapidly in real-time. Geomagnetism and crowdsensing-powered indoor navigation (GROPING) is a system that involves the use of a map for localization and navigation [31]. It is based on magnetic fingerprinting as well as Wi-Fi fingerprinting using the Dijkstra algorithm [32] and Monte Carlo localization [33]. The component that builds the map incorporates all semantic information as well as all discovered landmarks that make the map easy to use. Its main advantage is the non-dependence on any infrastructure support. WiSLAM is a technology that uses two SLAM technologies, PlaceSLAM and FootSLAM, to track the user’s location [34,35,36]. WiSLAM uses landmarks effectively by utilizing each SLAM according to the type of landmark. However, since the two SLAM technologies are used simultaneously, the computation time increases, which makes it difficult to apply the calculation process to mobile devices. SemanticSLAM has the robustness for use in any building other than a specific building using the landmark retrieved from the user’s sensor and the landmark simultaneously generated from the indoor RSS value [37]. In addition, SematicSLAM can reduce the time required to create a landmark map by generating one of the two landmarks in advance if the interior information is known. However, there is a problem that the SemanticSLAM does not perform the update of the landmark separately, so even if the landmark is no longer being used, it is not removed. The robust crowdsourcing-based indoor localization system (RCILS) is an indoor location tracking system that calibrates a user’s position using his movement data according to the indoor structure and RSS data of a corresponding location as landmarks [38]. RCILS predicts indoor movement using sensor data and the user’s indoor map and combines these with observation result from Wi-Fi to generate landmarks. However, there exists a problem in that it is difficult to apply RCILS to all buildings because a map is required to acquire room information in advance. Phillips has proposed the visible light communication (VLC) [39,40] based indoor positioning system which uses light from LED luminaires and does not require additional installations. The system locates a user by sending a unique code to a mobile device. The advantages of the solution are that it is empowered by iPhone operating system (iOS), Android software development kit (SDK) as well as cloud services, which allow third-party developers to incorporate the positioning capabilities into their customizable application. Yuanchao Shu et al. developed G-Loc [41], a gradient-based fingerprint map system that uses a map (Gmap [42]) and then leverages RSSI values to establish the user location using the extended particle filter algorithm. Although it can reduce the cost associated with fingerprint map calibration, its performance can be hampered by unexpected fluctuations of RSSI values. Chen, Zhenghua et al. propose a fusion approach of Wi-Fi, smartphone sensors and landmarks using the Kalman filter for indoor localization [43]. They use the weighted path loss (WPL) algorithm to track the location of Wi-Fi and conducts pedestrian dead reckoning (PDR) using smartphone sensors. They use also the knife-only filter to fuse the two techniques. Without the direct Wi-Fi installation in the building, it is difficult to pinpoint the location and the number of Wi-Fi installed in the building. Zou, Han et al. propose an accurate indoor positioning algorithm using mobile phone inertial sensors, Wi-Fi and iBeacon [44]. They use inertial measurement unit sensors for PDR and use Wi-Fi fingerprinting and iBeacon by sensor fusion based on a particle filter for corrections. Not only does this technology require both infrastructure to be installed indoors, but also the Wi-Fi’s desired use requires upgrading the router’s firmware.

The aforementioned existing indoor positioning technologies attempt to correct errors of sensor data using auxiliary sensors, combining several algorithms, or diversifying landmarks. However, the problem with using auxiliary sensors is that these sensors can only be used in limited environments. For example, in the case of BatTracker which uses sound waves, it cannot be used in large halls or places with many objects. When using composite algorithms, it is impossible to correct sensor errors quickly due to decreased calculation speed in Smartphones, which have lower processing power than normal central processing units (CPUs). In the case of techniques that diversify landmarks, they only consider the addition of landmarks and do not consider the deletion of unnecessary landmarks. Our proposed indoor positioning technique, IPSCL attempts to address these problems. IPSCL utilizes only the built-in basic sensors of smartphones and accurately finds the position of a user using the gyroscope sensor and the magnetometer sensor alternatively, based on characteristics of their values. IPSCL also enables rapid location correction using the Bluetooth beacon-based landmark approach. Finally, IPSCL allows users to add crowdsourced landmarks which in turn can be used. It also updates the number of detection of crowdsourced landmarks and spreads most recent and most frequently used crowdsourced landmarks to users.

## 3. Indoor Positioning with Sensors and Crowdsourced Landmarks (IPSCL) System Design

**Figure 1:**
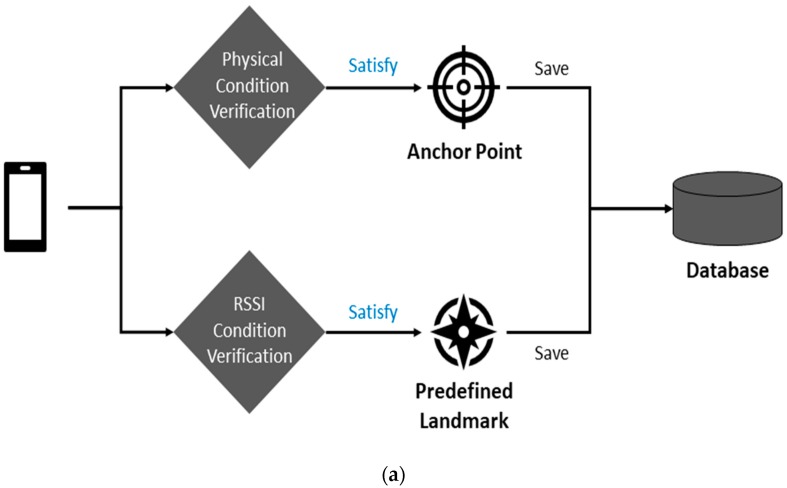

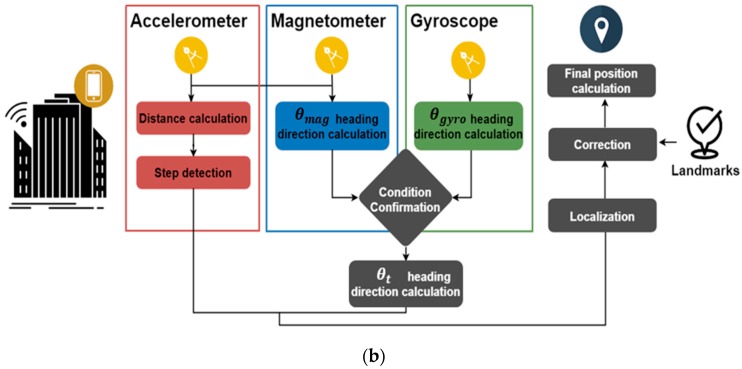
Indoor positioning with sensors and crowdsourced landmarks (IPSCL) system architecture (**a**) offline anchor points and predefined landmarks setup phase (**b**) localization phase.

This section describes the algorithm of our proposed indoor positioning technique, IPSCL, which consists of two sub-phases. Figure 1 presents the system architecture. In the offline anchor points and predefined landmarks setup phase, IPSCL builds up a list of anchor points and a list of predefined landmarks. The anchor point is considered as the starting point of indoor positioning, such as the entrance of a building, and the landmark is the position to be considered as the reference point. In the localization phase, IPSCL calculates the position of a user using the gyroscope and the magnetometer alternatively, based on characteristics of their values. IPSCL also corrects the position of users based on landmarks and allows users to add the location where they repeatedly detect the same and special beacon RSSI values as a crowdsourced landmark.

### 3.1. Offline Anchor Points and Predefined Landmarks Setup Phase

We install Bluetooth beacons evenly [45,46] in the indoor spaces of interest for the anchor point and the landmark. Firstly, IPSCL selects the anchor point in the indoor environment. The anchor point is the physical location which is expected for users to start the localization. IPSCL sets locations such as entrances of the building, elevators, and stairs as anchor points. The *i*th anchor point *a_i_* has the following properties: (*x_a_i__*, *y_a_i__*, *BList_a_i__*). (*x_a_i__*, *y_a_i__*) is the fair of coordinates of *a_i_*, that are decided in advance. *BList_a_i__* is the list of Bluetooth beacons with RSSI values that are detected at *a_i_*. ${BList}_{a_{i}}$ has the following properties {($ID_{B1}$, $RSSI\_Value_{B1}$), ($ID_{B2}$, $RSSI\_Value_{B2}$)…}, where the $ID_{Bi}$ of the *i*th Bluetooth beacon and ${RSSI\_Value}_{Bi}$ is the RSSI value of the *i*th Bluetooth beacon. IPSCL stores the information of the anchor points in the server.

When IPSCL user enters the building and the GPS signal disappears, he/she measures the RSSI values of Bluetooth beacons in the current position and receives a list of anchor points from the server. IPSCL searches the nearest anchor point which has similar AP lists by the k-nearest neighbor (KNN) algorithm [47,48]. Once IPSCL user finds the nearest anchor point, she/he selects the location as the starting point of indoor positioning and starts the indoor positioning.

**Figure 2:**
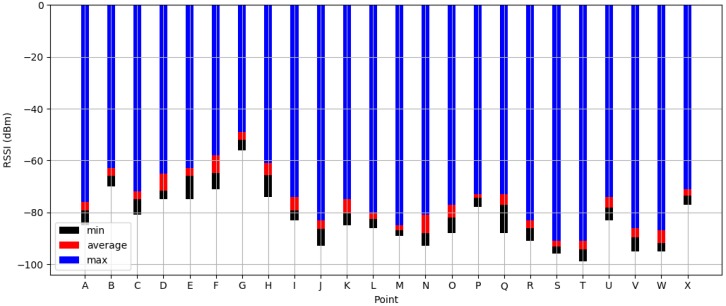
Experimental results of the process whereby predefined landmarks are found.

Secondly, IPSCL selects predefined landmarks in the indoor environment. The landmark is a datum point, which can be visible to serve as a reference by being differentiated from other points and showing consistent patterns. IPSCL sets as predefined landmarks, locations where RSSI values of Bluetooth beacons are constant and special in the repeated measurements. Figure 2 presents an example of experimental results of the process by which predefined landmarks are founded. The predefined landmark must receive at least one signal larger than −80 dBm on average and the difference between its maximum and minimum is smaller than 7 dBm during the 10 measurements. Locations B, G, P, and X are set to the predefined landmarks. Attributes of the predefined landmark are similar to those of the anchor point. Anchor points can also be included in predefined landmarks if they have characteristics as landmarks. However, the anchor point is different from the predefined landmark. The anchor point is the physical location, such as entrances of the building, elevators, and stairs, expected for users to start the localization. The predefined landmarks are defined to the location receiving beacon signals with large and constant RSSI values.

Where there is a lack of the network connectivity, the IPSCL user cannot use either the anchor point or the predefined landmark and its performance will be degraded. This problem can be solved by using an offline-first mobile application, where IPSCL user knows the anchor points and the predefined landmarks beforehand.

### 3.2. Localization Phase

#### 3.2.1. Built-In Sensor-Based Localization

Once the starting point is determined by searching anchor points, the system uses the built-in sensors of the smartphone to measure the movement of the user. Firstly, IPSCL detects the steps of the user using the step counter. The step counter is used to confirm whether the user has moved and this value is determined by measuring the change of the acceleration sensor’s readings. When IPSCL detects the step of a user, it calculates two values for the distance and the heading direction. The distance is the value that confirms how long the user moves. The heading direction is the value that confirms which direction the user is heading.

In time t, the system calculates distance $d_{t}$ using the value of the acceleration sensor $\alpha_{t}$ according to the following equation:

(1) $$d_{t}=\int_{t-1}^{t}\left(\int_{t-1}^{t}\alpha_{t}dt\right)dt$$

IPSCL calculates the value of the current heading direction $\theta_{t}$ using the values of the previous heading direction $\theta_{t-1}$ using data from the magnetometer sensor and the gyroscope sensor.

$\theta_{t}^{mag}$ is calculated from the values of the accelerometer and the magnetometer sensors. When IPSCL detects a change in the sensor readings, it measures the following values:

(2) $$Val^{accel}=\{x^{accel},y^{accel},z^{accel}\}$$

(3) $$Val^{mag}=\{x^{mag},y^{mag},z^{mag}\}$$

where x, y, and z represent the sensor values measured on the corresponding axis of the smartphone respectively. Given that these values are measured relative to the smartphone, IPSCL converts them from the device coordinates system to the coordinate system defined as a direct orthonormal base. IPSCL calculates the following 3∗3 rotation matrix R [47,48].

(4) $$R=\left[\begin{array}{ccc} R_{00} & R_{01} & R_{02}\\ R_{10} & R_{11} & R_{12}\\ R_{20} & R_{21} & R_{22} \end{array}\right]$$

The first row of matrix R is calculated using values of the accelerometer and the magnetometer sensors as follows:

The third row of matrix R is calculated only using values of the accelerometer sensor as follows:

(5) $$\{\begin{array}{c} R_{20}=x^{accel}*num_{2}\\ R_{21}=y^{accel}*num_{2}\\ R_{22}=z^{accel}*num_{2} \end{array}$$

(6) $$num_{2}=\frac{1}{\sqrt{{\left(x^{accel}\right)}^{2}+{\left(y^{accel}\right)}^{2}+{\left(z^{accel}\right)}^{2}}}$$

Finally, the second row of matrix R is calculated using the first and third rows as follows:

(7) $$\{\begin{array}{c} R_{10}=R_{21}*R_{02}-R_{22}*R_{01}\\ R_{11}=R_{22}*R_{00}-R_{21}*R_{02}\\ R_{12}=R_{20}*R_{01}-R_{21}*R_{00} \end{array}$$

When the rotation matrix is completed, IPSCL calculates $\theta_{t}^{mag}$ based on the following equation:

(8) $$\theta_{t}^{mag}=atan2\left(R_{00},R_{11}\right)$$

$\theta_{t}^{gyro}$ is calculated from values of the accelerometer and the magnetometer sensors. When the smartphone detects a change in sensor readings, it measures $Val_{t}^{gyro}$, which is the value of the gyroscope sensor. $Val_{t}^{gyro}$ is the speed value that indicates how fast the heading direction changes with time t. IPSC calculates the variable value $\Delta \theta^{gyro}$ using the following equation:

(9) $$\Delta \theta^{gyro}=\int_{t-1}^{t}Val_{t}^{gyro}dt$$

Given that $\theta_{t}^{gyro}$ is determined by adding the angle change of the previous rotation sensor reading to the current, it is calculated as follows.

(10) $$\theta_{t}^{gyro}=\theta_{t-1}^{gyro}+\Delta \theta^{gyro}$$

**Figure 3:**
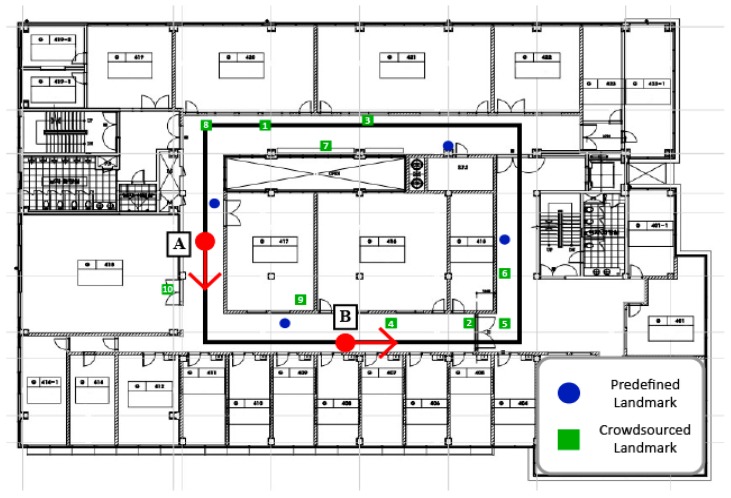
Indoor layout of testbed building with ground-truth reference path.

**Figure 4:**
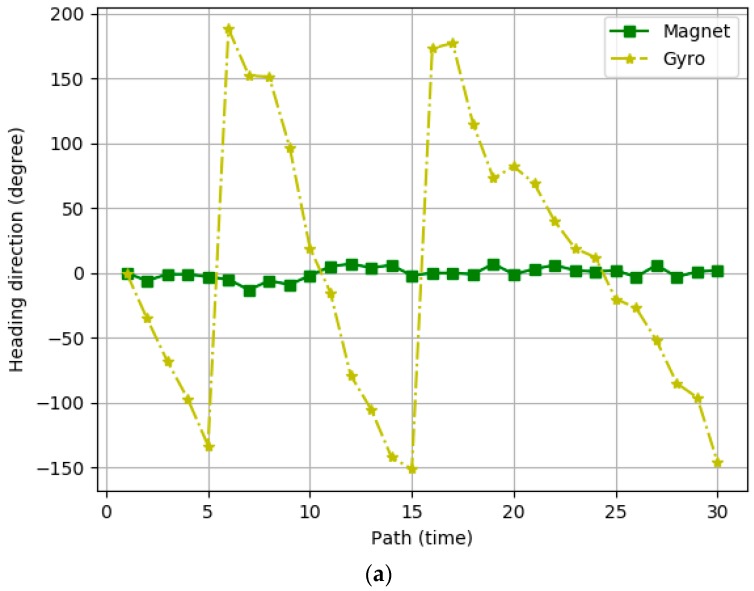

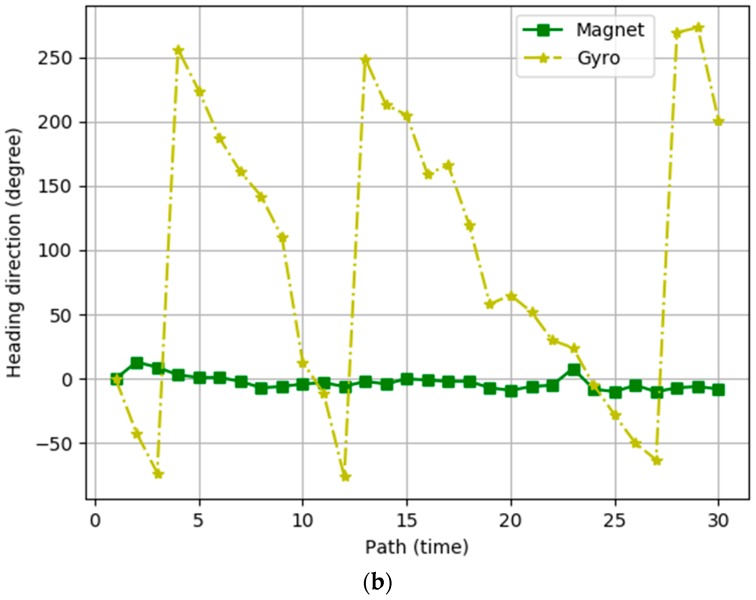
Heading directions measured at (**a**) the point A and (**b**) the point B in Figure 3.

To examine the properties of the heading direction values calculated using the gyroscope and magnetometer sensors, we performed experiments whereby a user measured his position 30 times using either the magnetometer sensor or the gyroscope sensor while walking along the path shown in Figure 3. Figure 4 presents the heading directions measured at points A and B of Figure 3 as a function of the number of paths for the gyroscope sensor and the magnetometer sensor respectively. The values of the magnetometer sensor are almost constant in the same location, but the values of the gyroscope sensor fluctuate. Based on these experimental results, we conclude that the heading direction values of the magnetometer sensor are generally correct, and we argue that the accurate heading directions of moving users must be calculated using the gyroscope sensor and the magnetometer sensor alternatively, according to the characteristics of their values. In the results, IPSCL calculates the heading direction using the following equation based on the values of the two sensors $\theta_{t}^{gyro}$ and $\theta_{t}^{mag}$

(11) $$\{\begin{array}{c} R_{00}=\left(y^{mag}*z^{accel}-z^{mag}*y^{accel}\right)*num_{0}\\ R_{01}=\left(z^{mag}*x^{accel}-x^{mag}*z^{accel}\right)*num_{0}\\ R_{02}=\left(x^{mag}*y^{accel}-y^{mag}*x^{accel}\right)*num_{0} \end{array}$$

(12) $$num_{0}=\frac{1}{\sqrt{{\left(y^{mag}*z^{accel}-z^{mag}*y^{accel}\right)}^{2}+{\left(z^{mag}*x^{accel}-x^{mag}*z^{accel}\right)}^{2}+{\left(x^{mag}*y^{accel}-y^{mag}*x^{accel}\right)}^{2}}}$$

where $l^{\theta }$ is the limit of $\Delta \theta^{gyro}$ which means that there is a significant change in the gyroscope sensor. The system calculates the heading direction according to three conditions. In the first case, $\Delta \theta^{gyro}$ is greater than $l^{\theta }$, and the magnetometer value, $\theta_{t}^{mag}$, is smaller than the sum of the previous heading direction, $\theta_{t-1}$, and $\Delta \theta^{gyro}$. This means that there are meaningful rotations and the magnetometer sensor works well. IPSCL calculates the heading direction with $\theta_{t}^{mag}$. In the second case, if $\Delta \theta^{gyro}$ is greater than $l^{\theta }$ but $\theta_{t}^{mag}$ is greater than the sum of $\theta_{t-1}$ and $\Delta \theta^{gyro}$, this means that there are meaningful rotations but the magnetometer sensor is affected by the surrounding environments. In this case, IPSCL calculates the heading direction with $\theta_{t}^{gyro}$. Finally, if $\Delta \theta^{gyro}$ is lower than $l^{\theta }$, which means there is no significant rotation. Therefore, IPSCL calculates the heading direction with $\theta_{t-1}$.

(13) $$\theta_{t}=\{\begin{array}{c} \theta_{t}^{mag},(\theta_{t-1}+\Delta \theta^{gyro}>\theta_{t}^{mag},\Delta \theta^{gyro}>l^{\theta })\\ \theta_{t}^{gyro},(\theta_{t-1}+\Delta \theta^{gyro}\leq \theta_{t}^{mag},\Delta \theta^{gyro}>l^{\theta })\\ \theta_{t-1},\left(\Delta \theta^{gyro}\leq l^{\theta }\right) \end{array}$$

After IPSCL calculates the distance and the heading direction, it calculates the coordinates of the user. The system calculates the current coordinates $\left(x_{t},\;y_{t}\right)$ according to the following equation:

(14) $$\{\begin{array}{c} x_{t}=x_{t-1}+\Delta x\\ y_{t}=y_{t-1}+\Delta y \end{array}$$

Δx and Δy are calculated using the sine and cosine of $\theta_{t}$ as follows:

(15) $$\{\begin{array}{c} \Delta x=d_{t}*cos\left(\theta_{t}\right)\\ \Delta y=d_{t}*sin\left(\theta_{t}\right) \end{array}$$

#### 3.2.2. Predefined and Crowdsourced Landmark-Based Correction

In the built-in sensor-based localization, IPSCL corrects the user’s heading direction, but the user’s measured location may be inaccurate because it is calculated using the previous location. Given that there is no reference point, the measured location may be erroneous and may be incorrectly accumulated. IPSCL corrects these errors based on landmarks. Algorithm 1presents the pseudo-code of the landmark algorithm. Whenever IPSCL calculates the location of the user, it checks the value of isDetected and distances between the calculated location and all landmarks. IsDetected has the Boolean value which helps to check if the landmark was detected immediately before. If isDetected value of a landmark is true, it means IPSCL used that landmark immediately before. It prevents IPSCL from continuing to bind to one landmark. Distance is the physical distance between landmarks and the calculated location of the user. IPSCL calculates the distance with the following equation:

(16) $$distance=\sqrt{{\left(x^{lm}-x_{t}\right)}^{2}+{\left(y^{lm}-y_{t}\right)}^{2}}$$

**Algorithm 1.** Pseudo code of the landmark algorithm.// *Positioning*
*if(*
$isDetected^{lm}==false\;\&\&\;distance<l^{distance}$
*){*
   *if*$(isSimillar\left(BList^{t},BList^{lm}\right)\{$
      *set_AllLandmark_IsDetected(false)*    // Refresh used landmarks      *(*$x_{t},y_{t}$*) = (*$x^{lm}$*,*
$y^{lm}$)      $isDetected^{lm}$*= true*             // Prevent continuous capture *}*   *if*$\left(lm\in lm^{crowdsourced}\right)$
      $detection\_number^{lm}++$       // Use for setting transfer priority// *Insert*
*if*
$\left(BList^{t}\;satisfied\;condition\_lm\right)$
*{*
      $lm^{new}$
*= (*$x_{t},y_{t},BList^{t},false,0$*)*      *addAToB(*$lm^{new},lm^{crowdsourced})$
      *sendToServer(*$lm^{crowdsourced}$*)*       *}*// *Reset*
*if(periodIsCome){*
   *for n=1 to all_*$lm^{crowdsourced}$
      $detection\_number^{lm}$* = 0*          // Identify useless landmarks used a lot in past *}*

If the distance is smaller than $l^{distance}$, the limit which IPSCL use for judging close enough to landmark. If the isDetected value is false and the distance is smaller than $l^{distance}$, IPSCL compares the bluetooth RSSI list of current location and Bluetooth RSSI list of the relevant landmark. If two lists are similar, the calculated location forcibly moves to the location of the landmark. As the number of landmarks increases, the accuracy of IPSCL improves. IPSCL allows users to add crowdsourced landmarks locations where measured RSSI values of Bluetooth beacons are satisfied by the requirement of the predefined landmark. Predefined landmarks are considered credible. Crowded landmarks may not be credible but are worthy of reference, therefore the IPSCL server manages them separately. The IPSCL server manages the number of detections per crowdsourced landmark. Whenever an IPSCL user finds a new landmark, he/she sends the crowdsourced landmark to the IPSCL server. IPSCL server adds it to the list of crowdsourced landmarks and then sends them to the IPSCL users whenever they are updated. When an IPSCL user detects one of the crowdsourced landmarks, this individual notifies the IPSCL server which then increments the number of detections for the crowdsourced landmark. The IPSCL server periodically sends to IPSCL user the list of crowdsourced landmarks with the largest number of detection times. To erase old crowdsourced landmarks, the server periodically resets the detection number of crowdsourced landmarks to zero.

## 4. Experiment

### 4.1. Experimental Setup

We performed extensive experiments to evaluate our proposed indoor positioning technique, IPSCL. IPSCL users walked the path shown in Figure 3 30 times for each experiment and fixed the device in front of the chest to minimize sensor error. Blue circles are predefined landmarks which are used in all experiments by default. Green rectangles are crowdsourced landmarks and their numbers are the orders that they are added by users. We stored the user’s coordinates at eight points along the path. The experiments were performed at the Engineering building of Sangmyung University. The size of the place was 14.88 m × 21.86 m (325.27 m^2^). On average, it took 57 s to walk the path. A Galaxy A5 (2017) device was used for the experiment, which included an acceleration sensor, a rotation sensor, and a magnetic sensor. For Bluetooth, we use a MiniBeacon with a 3V CR2477 battery. We developed IPSCL in an Android studio (SDK: 26) development environment, and the server managed landmarks using the database, PostgreSQL.

To evaluate IPSCL extensively, we compared the performance of IPSCL with those of the following five different approaches. The first is the indoor positioning technique using the magnetometer sensor of the smartphone, called IPmag. The second is the indoor positioning technique using the gyroscope sensor of the smartphone, called IPmag. The third is the indoor positioning technique using the magnetometer sensor and the gyroscope sensor of the smartphone, called indoor positioning with sensors (IPS). The fourth is the indoor positioning technique using the magnetometer sensor and the gyroscope sensor of the smartphone and predefined landmarks, called IPSPL. Our proposed technique, IPSCL uses the magnetometer sensor and the gyroscope sensor of the smartphone, predefined landmarks and crowdsourced landmarks. The fifth is the IPSCL with indoor map information, called IPSCLmap. IPS, IPSPL, and IPSCL are our own techniques which were described in Section 3, and IPmag and IPgyro are provided as references for the performance comparison. IPSCLmap is also provided as a reference to show that our proposed mechanism can be more improved in a typical situation where the indoor map is known by ruling out the unreachable areas. The algorithm we applied as follows. When estimated user’s position is an unreachable area, IPSCLmap determines that the user’s heading direction is wrong. IPSCLmap changes the heading direction by 1° and calculates the new position using the changed direction and the previous position. At this point, IPSCLmap changes the direction at the same time to left and right. IPSCLmap selects as the heading direction the direction which finds the reachable area faster. IPSCLmap then continues the position using the newly calculated heading direction and position.

### 4.2. Experimental Results

#### 4.2.1. Heading Direction Error

**Figure 5:**
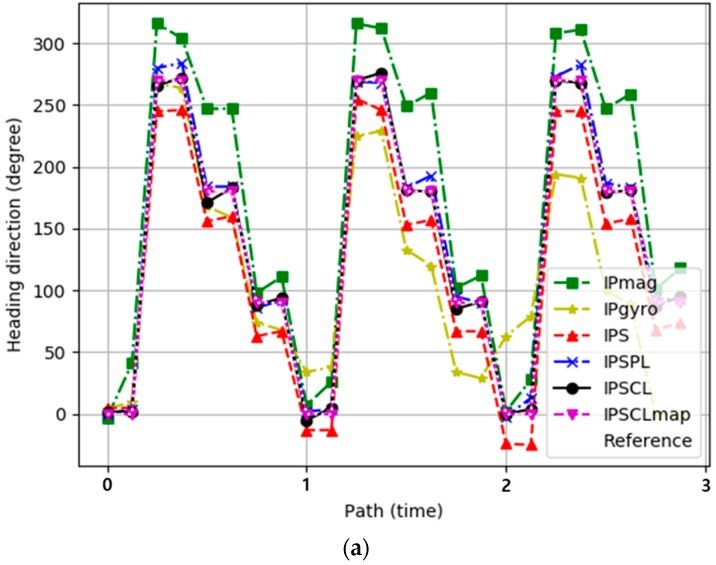

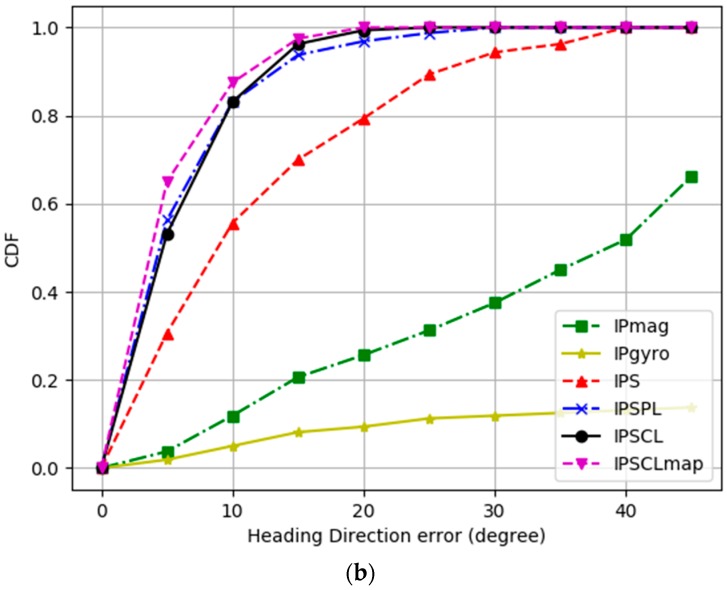
(**a**) Heading direction as a function of the path; (**b**) cumulated heading direction error for the ground-truth reference heading direction.

Figure 5a represents the experimental results for the heading direction estimation error for IPmag, IPgyro, IPS, IPSPL, IPSCL, and IPSCLmap. The *x*-axis represents the number of turns to the path and the *y*-axis indicates the heading direction. The black dotted line is the heading direction of the ground-truth reference path. The heading direction of IPmag was very different from the ground-truth reference direction, but the difference was similar as the number of paths increased. This is because the heading direction of IPmag is constant as shown in Figure 4. The heading direction of IPgyro was similar to the reference direction at the beginning of the path, but the difference increased as the number of paths increased. This is because the heading direction of IPgyro is calculated based on the previous heading direction and errors accumulate as shown in Figure 4. IPS showed much better performance than both IPmag and IPgyro. This is because IPS improves the accuracy of the heading direction mixing the magnetometer sensor and the gyro scope sensor. IPSPL showed better performance than IPS, IPSCL showed better performance than IPSPL, and PSCLmap exhibited the best performance. Table 1 shows the maximum, minimum, and average heading direction error of all techniques.

Figure 5b represents the experimental results for the cumulated heading direction error for IPmag, IPgyro, IPS, IPSPL, IPSCL, and IPSCLmap. The *x*-axis represents the heading direction error. This error is the difference between the ground-truth reference heading direction and the heading direction for a given technique. A total of 80% of the heading direction errors were smaller than 50 degrees in IPmag, they were smaller than 20° in IPS, and they were smaller than 10° in both IPSPL and IPSCL. The heading direction error of IPgyro was much worse than those of other techniques. In terms of heading direction error, IPS was better than IPmag and IPgyro, IPSPL and IPSCL were better than IPS, and IPSCL exhibited similar performance to IPSPL. IPSCLmap exhibited the best performance by ruling out unreachable area. The experimental results show that landmarks are effective for indirectly correcting the estimation of the heading direction, although they do not correct the estimation of the heading direction directly.

#### 4.2.2. Distance Error

**Figure 6:**
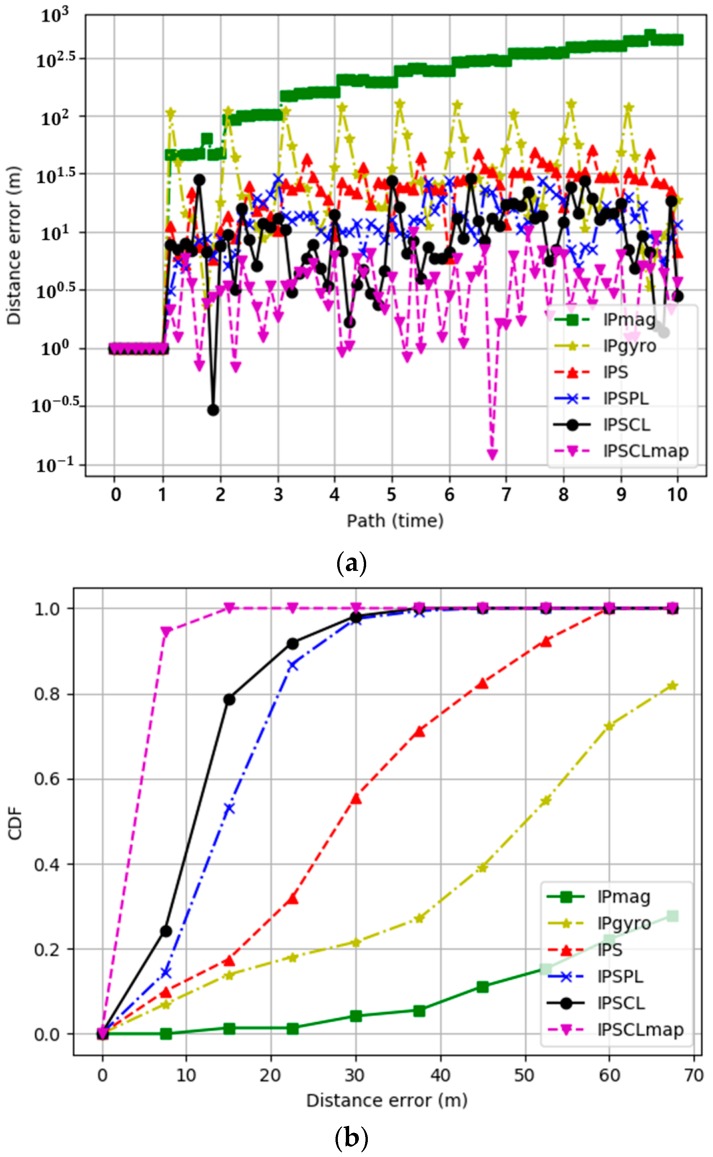
(**a**) Distance error for the ground-truth reference path as a function of the path; (**b**) cumulated distance error for the ground-truth reference path.

Figure 6a represents the experimental results for the distance error for the ground-truth reference path for IPmag, IPgyro, IPS, IPSPL, IPSCL, and IPSCLmap. The x-axis represents the number of paths. The *y*-axis was displayed as the log scale, because differences of values of methods were highly different. The distance error of a technique is the difference between the ground-truth reference path and the technique distance. In first path, all of techniques show similar paths to the ground truth path, so distance errors were almost 0. After first path, errors occur and values change, where the distance error may look suddenly increasing, but it is not actually. Because the *y*-axis is the logarithmic scale, the variations look large, and they are not large actually. Table 2 shows the maximum, minimum, and average distance error of techniques. The experimental results show that IPS and the landmarks help to reduce the distance error. Crowdsourced landmarks of IPSCL are more effective than IPSPL because IPSCL facilitates a greater number of landmarks than IPSPL. IPSCLmap exhibited the best performance by ruling out unreachable area. Table 2 shows the maximum, minimum, and average distance error of all techniques.

Figure 6b represents the experimental results for the cumulated distance error for the ground-truth reference path for IPmag, IPgyro, IPS, IPSPL, IPSCL, and IPSCLmap. The x-axis represents the distance error. A total of 80% of the distance errors were smaller than 65 m for IPgyro, they were smaller than 43 m for IPS, and they were smaller than 22 m and 15 m for IPSPL and IPSCL respectively. The distance error of IPmag was much worse than those of other techniques. In the case of IPS, its distance error was much smaller than both the IPmag and IPgyro, but once an error occurs, it is accumulated and increases because there was no reference point. IPSPL helps to reduce the distance error; however, IPSCL is more effective than IPSPL. PSCLmap exhibited the best performance by ruling out unreachable area.

#### 4.2.3. Crowdsourced Landmark

**Figure 7:**
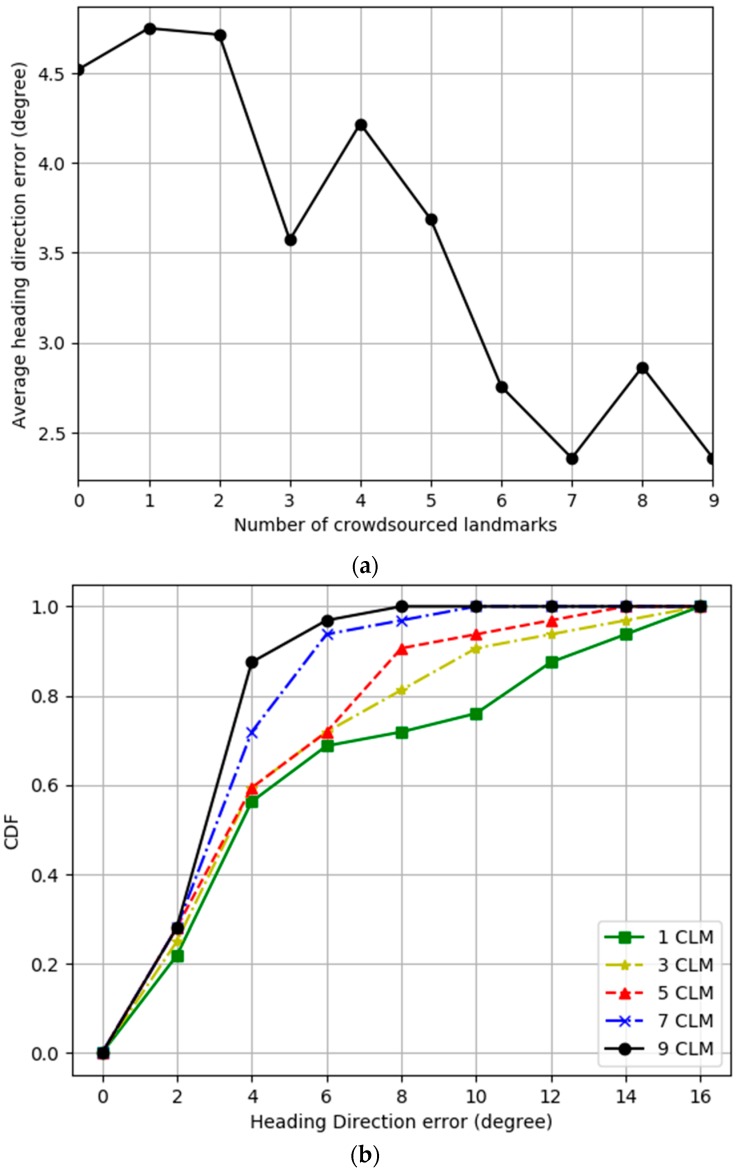
(**a**) Average heading direction error as a function of the number of crowdsourced landmarks (**b**) Cumulated heading direction error for the ground-truth reference heading direction for varying number of crowdsourced landmarks.

**Figure 8:**
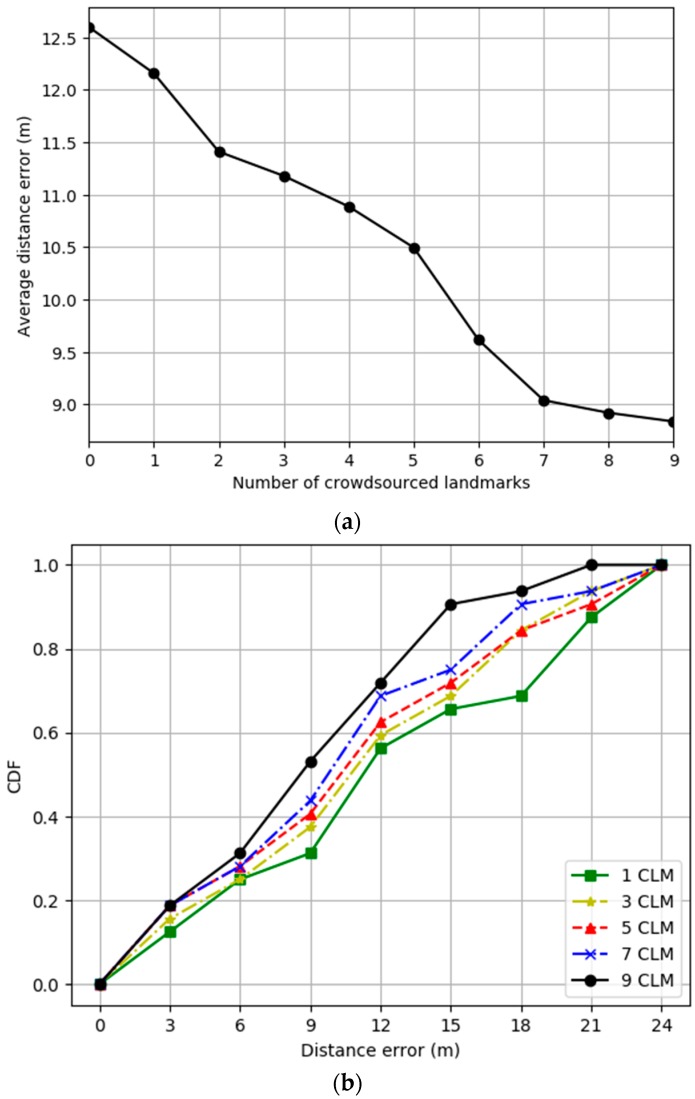
(**a**) Average distance error as a function of the number of crowdsourced landmarks; (**b)** cumulated distance error for the ground-truth reference path for varying number of crowdsourced landmarks

Figure 7 and Figure 8 present experimental results varying number of crowdsourced landmarks, where predefined landmarks are used in all experiments by default. Figure 3 shows the order that crowdsourced landmarks are added. Figure 7a represents the experimental results for the average heading direction error as a function of the number of crowdsourced landmarks for IPSCL. The *x*-axis represents the number of crowdsourced landmarks. As the number of crowdsourced landmarks increased, the average heading direction error decreased from 4.5 to 2.5°. Figure 7b represents the experimental results for the cumulated heading direction error for the ground-truth reference heading direction for varying number of crowdsourced landmarks for IPSCL. As the number of crowdsourced landmarks increased, the average heading direction error decreased.

Figure 8a represents the experimental results for the distance error as a function of the number of crowdsourced landmarks for IPSCL. The *x*-axis represents the number of crowdsourced landmarks. As the number of landmarks increased, the average distance error decreased from 12.2 to 8.78 m. Figure 8b represents the experimental results for the cumulated distance error for the ground-truth reference path for varying number of crowdsourced landmarks for IPSCL. As the number of crowdsourced landmarks increased, the average distance error decreased.

#### 4.2.4. Localization Trajectories

**Figure 9:**
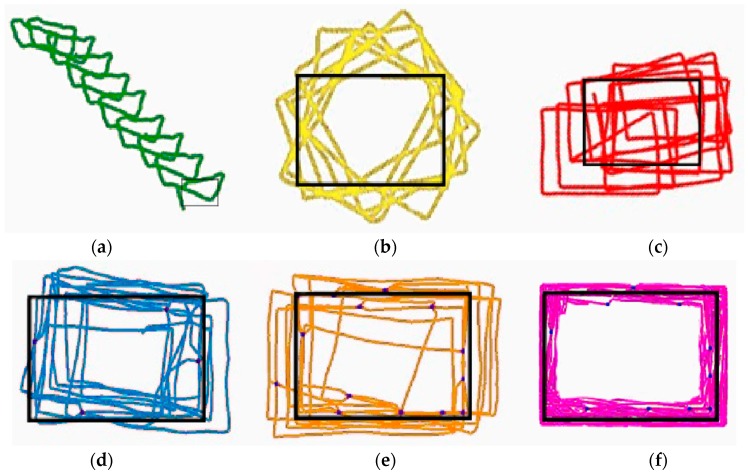
Localization trajectories for the ground-truth reference path for (**a**) IPmag (**b**) IPgyro (**c**) IPS (**d**) IPSPL (**e**) IPSCL (**f**) IPSCLmap.

Figure 9 represents the experimental results for the localization trajectories for IPmag, IPgyro, IPS, IPSPL, IPSCL, and IPSCLmap. The black dotted rectangle represents the ground-truth reference path. The blue dots are points of the landmarks. Localization trajectories of IPmag were similar to those of the ground-truth reference path in terms of the shape, but they increasingly got away from the ground-truth reference path as the number of the path increased. On the other hand, localization trajectories of IPmag warp more and more as the number of the path increased. IPS calibrates the heading direction of the smartphone well by mixing values of the magnetometer and gyroscope sensors, but its localization trajectories are occasionally far from the ground-truth reference path. This is because IPS does not have any reference points for indoor positioning. The localization trajectories of IPSPL are more similar to the ground-truth reference path than IPS. IPSCL has a greater number of landmarks than IPSPL and it exhibits the better performance. IPSCLmap exhibited the best performance by ruling out unreachable area.

## 5. Conclusions

Individuals spend most of their time in indoor spaces and as such, the importance of indoor positioning technique increases. The built-in sensors of a smartphone have the advantage in that they can be used for indoor positioning without installing any additional device separately. However, the readings of built-in sensors fluctuate and are occasionally different from each other, which impacts the accuracy of indoor positioning. Moreover, once an error occurs, the error can accumulate because there is no reference point for indoor positioning. In this paper, we present an accurate sensor-based indoor positioning technique that estimates accurate heading directions of the smartphone by mixing the readings of the gyroscope and magnetometer sensors based on their characteristics. To provide reference points for localization, we extended our proposed technique using landmarks. Our approach also allows users to add a crowdsourced landmark of the location where they repeatedly detect the same and special beacon RSSI values. We compare our proposed technique (sensors, predefined landmarks, and crowdsourced landmarks) with sensor-only techniques, sensors, and predefined landmarks techniques. Extensive experimental results show that the proposed technique facilitates the acquisition of accurate heading direction and coordinates of the user. We also extend our proposed technique to consider a typical situation that the indoor map is known and showed our proposed mechanism can be more improved by ruling out unreachable area.

## 6. Future Works

We have five ongoing future research works. First, our proposed technique includes crowdsourced landmarks. To the scalability, it must be able to support many number of users. Lots of connection between the server and users may be required to exchange the crowdsourced landmark information, which degrades the performance of our system. We believe that the effective communication protocol is an important issue in the crowdsourced situation that many users cooperate with each other. The push notifications can be a solution, and we need to apply it to our proposed technique. Second, quality of sensors is very different for various mobile devices, and sensor values may be different. We can solve this problem by raw data filtering and merging them with a Kalman filter [49,50,51]. Third, RSSI values may change in the presence of moving objects such as people in the building. It may cause an error. The landmark map must support such variation [52]. Fourth, the performance of our proposed technique can be improved in a typical situation where the indoor map is known by ruling out the unreachable areas. Although we provide experimental results, our proposed technique must be extended more effectively. Finally, indoor positioning is one of the most actively studied areas, and there are lots of state-of-art approaches. Of course, there are numberless fusion approaches [53]. Our proposed technique must be compared with state-of-art fusion approaches, though it is difficult to find corresponded approaches.

## Figures and Tables

**Table 1:** Maximum, minimum, and average heading direction error of techniques.

	IPmag	IPgyro	IPS	IPSPL	IPSCL	IPSCLmap
Maximum	83°	335°	35°	27°	23°	160°
Minimum	1°	3°	1°	1°	0°	0°
Average	38.76875°	127.8313°	11.10625°	5.56875	5.1625	4.2375

**Table 2:** Maximum, minimum, and average distance error of techniques.

	IPmag	IPgyro	IPS	IPSPL	IPSCL	IPSCLmap
Maximum	450.3055 m	127.753 m	58.12365 m	37.86159 m	33.21654 m	10.2354 m
Minimum	45.71716 m	2.330403 m	3.889214 m	0.649444 m	0.299748 m	0.120128 m
Average	224.1201 m	38.24919 m	29.12765 m	13.21881 m	10.95822 m	3.74123 m
